# Thermal/Mechanical Characteristics Simulation Analysis of Solder Layer Damage in IGBT Modules

**DOI:** 10.3390/mi17070827

**Published:** 2026-07-10

**Authors:** Jianbo Zhou, Jibing Chen, Liang He, Hui Tang, Xiaohu Wu

**Affiliations:** 1 School of Mechanical Engineering, Wuhan Polytechnic University, Wuhan 420023, China; 2 School of Mechanical Engineering, Sichuan University, Chengdu 610065, China; 3 School of Materials and Energy, University of Electronic Science and Technology of China, Chengdu 611731, China; 4 School of Integrated Circuits, Hubei University, Wuhan 430415, China; 5 Yangtze Laboratory, Wuhan 430250, China

**Keywords:** IGBT module, finite element simulation, solder layer damage, thermal/mechanical property

## Abstract

The insulated gate bipolar transistor (IGBT) is widely applied in industrial fields such as rail transit, wind power generation, smart grids, and renewable energy. The temperature distribution, stress variation patterns, thermal performance, and modeling damage in the solder layer of IGBT modules under thermal and stress loadings have rarely been studied. This study first established a three-dimensional geometric model based on the actual dimensions of the IGBT module. A finite element model was successfully constructed for thermal/mechanical multi-physics coupled simulation based on the ANSYS Workbench platform to simulate the temperature, deformation trends, and stress distribution patterns of the solder layer in the IGBT module. Secondly, the solder layer defects of the IGBT module were categorized into five major types, and 37 sets of 3D models of IGBT with damaged solder layers were designed, followed by thermal/mechanical coupled simulation analysis for each. Finally, the influence of the void positions, sizes, and distribution types in the solder layer on the module temperature, heat dissipation path, and thermal stress was simulated during thermal cycling. The results showed that the highest stress at the edge of the solder layer is 6.2504 × 10^7^ Pa, the lowest junction temperature is 70.79 °C, and the average thermal stress is 1.2388 (m/m). The highest junction temperature reached 72.562 °C under central solder layer damage states as determined by a thermal/mechanical coupled simulation analysis of four different types of solder layer defects. This research provides a theoretical basis and reliable technical support for the anti-damage and failure of IGBT modules and high-power devices.

## 1. Introduction

With the continuous improvement of the performance and reliability requirements of insulated gate bipolar transistor (IGBT) modules in the power electronics industry, especially in cutting-edge fields such as new energy vehicles and smart grids, optimizing the performance of IGBT packaging, especially the solder layer, has become the key to improving the overall equipment efficiency and lifespan [1]. As a core material in the field of electronic packaging, chip solder has garnered widespread attention globally, particularly focusing on improving the reliability of solder and exploring new soldering materials [2,3].

Furthermore, the advancement of high-reliability solder remains a key area of interest, with investigations aimed at enhancing the thermal fatigue performance, creep resistance, and aging resistance of solder. Zhou et al. [4] employed electrochemical techniques and surface characterization methods to investigate how various alloying elements influence the corrosion performance of Sn-Zn solder. The experimental results showed that the addition of Ti, Ni, Cr, Cu, and Ag will enhance the stability of the passive film and refine the zinc-rich precipitates, thereby improving the corrosion resistance of Sn-9Zn alloy. Liu et al. [5] designed high-temperature tensile tests to study the mechanical properties of SAC solder doped at high temperature and conduct characterization. Yang et al. [6] compared three different types of low-temperature lead-free solder alloys based on Sn-Zn and Sn-Bi. The influence of elemental compositions on the solder’s properties was examined, with the technical standard being the surface mount technology (SMT) used in the production of electronic packaging. When comparing the variations in silver content within a Sn-Ag-Cu solder system, Xu et al. [7] made some high-silver-content solders with a higher concentration of intermetallic compounds (Ag_3_Sn), and worse impact resistance was generated. Chen et al. [8,9] also simulated solder characteristics by designing a 3D IGBT module, which enhances the solder’s comprehensive performance level to that of leaded solder, while low-silver soldering materials solve these drawbacks. The future development of soldering materials will follow this theme and direction.

The sintering technology of solders, as an advanced interconnection process, has indeed been widely applied in IGBT power electronic devices due to its excellent thermal conductivity, high reliability, and long lifespan, replacing traditional solder. However, this technology also has limitations. Its main restrictions and drawbacks include high cost, complex process control, material and thermal expansion coefficient matching issues, safety risks of nano-silver particles, and control of sintering layer defects. Overall, both internationally and domestically, the research on chip solder materials is moving in the direction of being environmentally friendly, having high performance, and being adaptable to diverse application requirements [10,11]. These studies not only advanced the technology of soldering materials but also provided solutions for the failure of soldering materials caused by alternating stresses resulting from rapid thermal cycling in the field of electronic packaging. This is to meet the increasingly high performance requirements in modern electronic devices.

This study focuses on the temperature distribution, stress variation patterns, and failure destruction mechanisms of the solder layer in the IGBT module under thermal and stress loadings. The structure of this paper begins with the description of the experimental design and simulation in the second part. The third part presents the results and discussion of the multi-physics field coupling simulation analysis based on solder layer damage for IGBT. The fourth part sets out our conclusions on the findings and outlines future prospects. Based on the ANSYS (2024 R1) Workbench platform, a finite element model is constructed for a multi-physics field coupling simulation. The temperature, stress distribution patterns, deformation trends, and evolution patterns of the solder layer in the IGBT module under normal working conditions are analyzed, providing technical support for improving the reliability of IGBT modules and related high-power devices.

## 2. Experimental Design and Simulation

This study initially conducted simulations under a passive thermal cycling condition without external voltage or conduction current. The main purpose was to completely isolate the electrochemical effects caused by electrical stress and the additional temperature rise resulting from current self-heating through the control of a single variable. The goal was to accurately extract the thermal mechanical stress distribution pattern of the solder layer solely caused by the alternating environmental temperature. These basic data can serve as a calibration benchmark for subsequent multi-stress coupling simulations, avoiding the difficulty of physical mechanism decoupling when multiple variables are superimposed.

The initial operating conditions for this stage of simulation are set to a conventional constant-temperature drying environment. The effects of humidity diffusion and surface condensation have not yet been incorporated into the multi-physics field coupling solution. The main purpose is to first obtain the pure thermal/mechanical fatigue evolution law of solder joints in the drying reference environment, in order to provide a reference benchmark for the subsequent analysis of the superimposed complex environmental variables. This model has not yet achieved full coupling calculation of the thermal/hydraulic/physical fields, and cannot directly reproduce the accelerated aging process of solder joints under high humidity and condensation conditions. The predicted fatigue life of the solder joints obtained from the simulation will be slightly higher than the actual service life under high humidity conditions. In the future, by introducing a wet diffusion solution module and combining aging tests under different humidity gradients to calibrate parameters, the simulation accuracy in the environmental factor dimension will be further improved.

In this study, a thorough simulation analysis is used to investigate how solder layer damage affects the thermal/mechanical performance of IGBT modules. SAC305 solder is employed as an example, the simulation experiment creates 37 models of solder layer damage and applies a multi-physics coupled simulation technique to create a precise multi-physics model, to ensure the reliability and accuracy of the comparative simulation results, and the same material properties and power loads as those used in normal IGBT module simulations were applied to conduct simulation experiments on the 3D model with crack damage. With the thermodynamic and mechanical properties, ten reference values were chosen in the void rate range of 1–25%, and the depth of the crack was controlled between 8 and 12 mm [12,13,14]. This allowed for a thorough analysis of the impact of various solder layer damages on the overall reliability of the module, providing a theoretical foundation and a technical route for optimizing the IGBT module’s design.

### 2.1. Principles of Thermal/Mechanical Coupled Analysis

The finite element analysis method in ANSYS Workbench is based on the differential equations of thermal conductivity in heat transfer, and numerically calculates the relevant parameters of the 3D model and the matrix corresponding to the boundary conditions. The differential Equation (1) for thermal conductivity is described as follows:

(1) $$\rho c\frac{\partial T}{\partial t}=\phi \left(\frac{\partial^{2}t}{\partial x^{2}}+\frac{\partial^{2}t}{\partial y^{2}}+\frac{\partial^{2}t}{\partial z^{2}}\right)+\theta$$

where *ρ* is the density, *c* is the specific heat capacity, *t* is the temperature, *θ* is the heat generation rate of the IGBT chip, and *ф* is the thermal conductivity.

In this simulation study, it is assumed that the materials, stress state, and all stress components of each solder layer in the IGBT module are isotropic. According to the further derivation of the generalized Hooke’s law, the expression for the stress field is given as follows (2):

(2) $$\gamma_{xy}=\frac{\tau_{xy}}{G},\gamma_{yz}=\frac{\tau_{yz}}{G},\gamma_{zx}=\frac{\tau_{zx}}{G}$$

where $G=\frac{e}{2\left(1+\mu \right)}$, e is the Young’s modulus, *μ* is the Poisson’s ratio, and τ_xy_, τ_yz_, and τ_zx_ represent the shear stress on the three faces, respectively. γ_xy_, γ_yz_, and γ_zx_ represent the shear strain on the three faces, respectively.

Due to the differences in the thermal expansion coefficients between the various layers of materials, an increase in module temperature will cause an increase in thermal stress between the structural layers [15]. Under alternating temperature and stress conditions, the materials are prone to creeping fatigue and even deformation (such as bonding wire detachment, solder layer damage, etc.), which in turn increases the thermal resistance between the layers, leading to an overall increase in the junction temperature of the module [16]. Therefore, there is a significant thermal/mechanical coupled phenomenon within the IGBT module [8,9]. The coupled expressions for the thermal field and mechanical field in the X, Y, and Z directions are given as follows (3):

(3) $$\varepsilon_{x}=\frac{\partial u}{\partial x}=\frac{1}{e}\left[\sigma_{x}-\mu \left(\sigma_{y}+\sigma_{z}\right)\right]+\alpha \Delta T\;\varepsilon_{y}=\frac{\partial v}{\partial y}=\frac{1}{e}\left[\sigma_{y}-\mu \left(\sigma_{z}+\sigma_{x}\right)\right]+\alpha \Delta T\;\varepsilon_{z}=\frac{\partial w}{\partial z}=\frac{1}{e}\left[\sigma_{z}-\mu \left(\sigma_{x}+\sigma_{y}\right)\right]+\alpha \Delta T$$

where u, v, and w are the displacements; σ_x_, σ_y_, and σ_z_ are thermal stress; ε_x_, ε_y_, and ε_z_ are thermal strain; α is the coefficient of expansion; and ∆*T* is the temperature difference between the two moments.

### 2.2. Finite Element Model Construction and Parameter Setting

**Figure 1:**
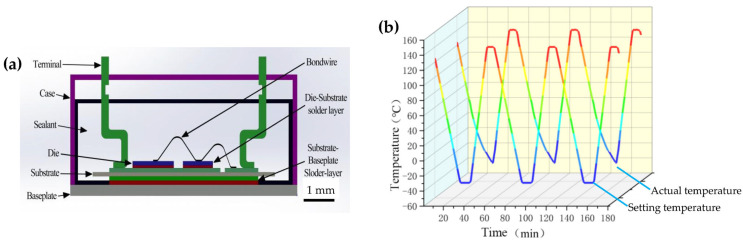
(**a**) Schematic diagram of IGBT internal structure [8]; (**b**) time/temperature profile of the solder layer in IGBT module [9].

This paper takes the conventional IGBT module as the research object, using SolidWorks software (2024 FD02) to establish a 1:1 thermal simulation model of the IGBT, as shown in Figure 1a. This model includes six layers of different structures, namely, IGBT chip and freewheeling diode (FWD), chip solder layer, substrate solder layer, direct bond copper (DBC), ceramic layer, and copper substrate, with specific parameters as shown in Table 1.

The simulated thermal cycling test parameters of this study were set as follows: the temperature range is from −40 to 150 °C, the number of cycles is 1000 times, the dwell time is 10 min, the ramp-down rate is 6.6 °C/min, and the rising rate is 11 °C/min. The design of this experiment refers to the standard GB/T 2423.22-2012 Environmental Testing Part 2 and the test method JESD22-A104 Temperature Cycling [9,17,18]. The specific time/temperature profile is shown in Figure 1b. All the test results in this manuscript were extracted during the same period of high-temperature dwell. All the figures and related discussions in this manuscript follow this standard.

Due to the differences between the simulation process and actual working conditions, to highlight the focus of this study and reduce experimental errors to improve the accuracy of the conclusions, the following settings were made for this simulation process, taking into account the computer’s operating speed and memory capacity:

(1) When conducting damage simulations, circular non-through weld holes are used to study the effects of porosity and pore radius changes on junction temperature. To ensure the accuracy of the simulation results, the porosity in the solder layer is replaced with air properties.
(2) The multi-discrimination method is used to mesh the module as a whole, and the mesh of the solder layer is encrypted according to the research focus. The meshing type is a hexahedral element, and the grid layers are divided into 3 layers along the thickness direction. The size of the elements is controlled at 0.1 mm. After simulation verification, the grid size is selected as 0.001 m, and the encryption grid size is chosen as 0.000009 m.
(3) During this simulation process, the forced convective heat transfer was applied to the bottom plate and the periphery to simulate the heat dissipation effect of the two components. The equivalent convective heat transfer coefficient of the substrate is 3000 W/m^2^·K^−1^, the convective heat transfer coefficient around the substrate is 10 W/m^2^·K^−1^, and the heat flux around the IGBT chip is 1500 W/m^2^.
(4) Using IGBT chips as the heat source and with uniform heat generation, ignoring the influence of heat radiation on heat transfer, the heat generation rate of the chips is determined. The chip’s power loss is set at 100 watts. The heat generation rate H = P/V = 8 × 109 W/m^2^·K^−1^, and a transient thermal simulation with a heating cycle of 40 s is conducted.

The specific parameters of the materials in the model are reported in reference [19], and these data are sourced from the Infineon official website and the material handbook.

## 3. Results and Discussion

**Figure 2:**
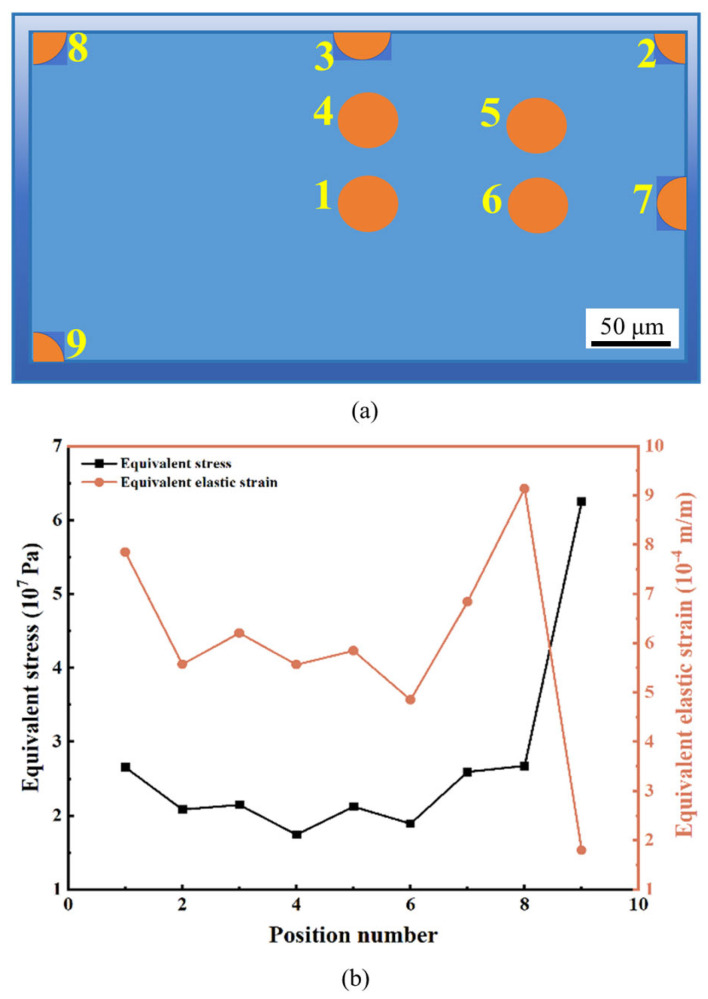
(**a**) Setting the void position number from 1 to 9 in the solder layer; (**b**) the curve of the solder layer equivalent stress and equivalent elastic strain at different positions.

To simulate the failure of IGBT modules due to extreme temperature cycling that may occur in practical applications, this paper selected nine representative void damage locations to simulate the possible defects in the solder layer, as shown in Figure 2a. These cavity settings reflect the real defects that may appear in the solder layer due to manufacturing defects, thermal cycling, or material aging. By simulating defect models at different locations, we can analyze the characteristics of module junction temperature changes and material stress changes caused by different types of damage, and predict material deformation. This allows for a better understanding and quantification of the impact of defects on module reliability [19].

The formation of solder layer damage is mainly due to two reasons [10]: 1. During the module soldering process, residual bubbles or incompletely evaporated reflow reactants in the solder can form voids in the middle of the solder layer [20]. 2. Due to long-term use, as the solder layer accumulates fatigue, voids of varying sizes can form and gradually expand [21]. Figure 2b shows the variation of equivalent stress and equivalent elastic strain from the center of the solder layer to the edge vertex. Due to the different temperature gradients, this variation curve is a repeated zigzag line. Overall, there is a trend of increasing stress toward the edges, with the maximum edge stress being 6.2504 × 10^7^ Pa, which is 2.4 times that at the center. This predicts that voids and cracks are likely to start from the edges and occur in the vertical direction of the chip, extending longitudinally, expanding laterally, and reaching toward the center. Therefore, when conducting thermal design for IGBT modules, the heat dissipation effect around the module should be given priority consideration to reduce the impact of thermal stress.

### 3.1. Finite Element Analysis Results of IGBT Modules with Cavitation Damage

**Figure 3:**
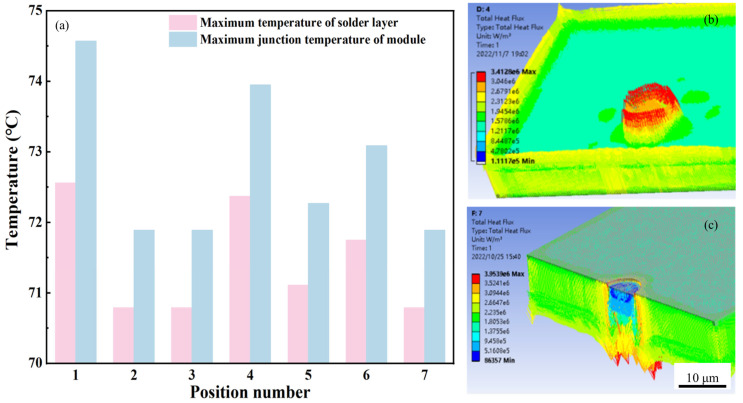
The junction temperature curve and thermal distribution of the module and solder layer in the IGBT at different void positions in the solder layer: (**a**) junction temperature curve; (**b**) thermal distribution of the solder layer at position 4; (**c**) thermal distribution of the solder layer at position 7.

**Figure 4:**
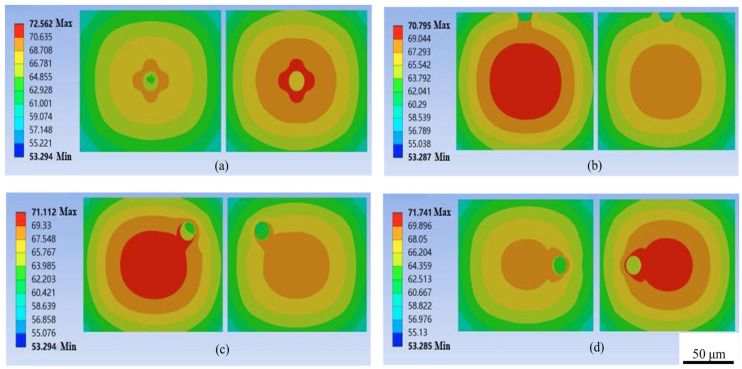
Cloud diagrams of IGBT solder layer junction temperature at different solder layer void positions: (**a**) position 1 [9]; (**b**) position 3; (**c**) position 5; (**d**) position 6.

The solder layer and overall thermal behavior of the IGBT module are significantly impacted by voids at various locations, as demonstrated in Figure 3b,c. The thermal distribution of the chip is impacted by voids because they change the local heat flow pathways. The results show that, apart from the central cavity, the position of the cavity can cause the high-temperature center of the chip to shift, altering the chip’s normal heat dissipation path [9,22,23]. Under the condition of a constant cavity size, the farther the cavity is from the heat source center, the lower the module’s highest junction temperature. With the least amount of damage, the voids at positions 2, 3, and 7 are closest to the solder layer’s edge. The junction temperature of the solder layer refers to the actual working temperature between the metals within the solder joint during the soldering process, which is usually higher than the external packaging temperature. As is shown in Figure 4, the temperature directly affects the reliability of the solder joint; if it is too high, it may cause problems such as metal oxidation and thickening of the IMC layer. It is the lowest at 70.795 °C (Figure 4b), which is close to the temperature of an undamaged module, and it has the least influence on the overall junction temperature when compared to other locations where the direction and magnitude of the heat flux are different. The damage at positions 1 and 4, particularly in the center of the heat source, is situated on the primary heat dissipation path. This makes the fatigue accumulation and heat accumulation more noticeable and has a greater effect on the module’s overall reliability. Consequently, since solder layer voids cannot be eliminated during production, efforts should be made to locate the voids as near to the substrate or chip’s edge as feasible [24,25,26]. This will lessen the chance of the IGBT module failing too soon by ensuring the stability of chip operation and the module’s dependability.

**Figure 5:**
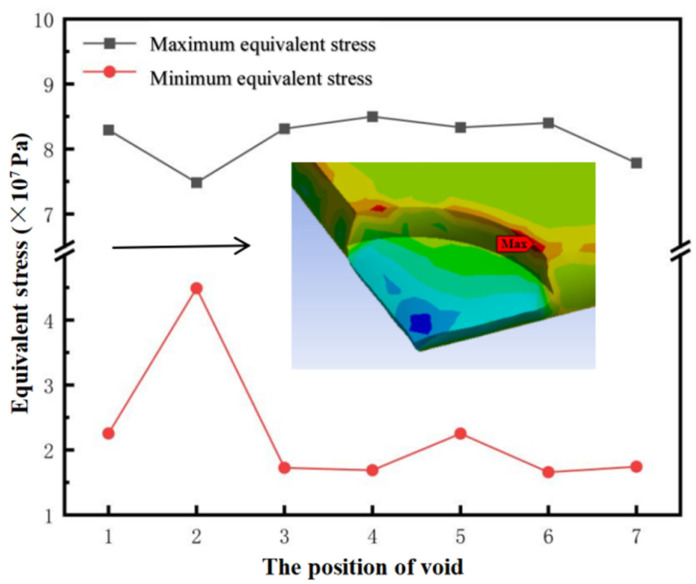
Equivalent stress of the solder layer at different void locations.

Material damage is primarily caused by changes in stress, and an excessively high equivalent stress can cause stress concentration in specific material areas. The IGBT solder layer’s maximum and minimum equivalent stress analysis results are displayed at seven different positions in Figure 5. The overall stress has increased to varying degrees as a result of the damage at various locations. Based on the previous analysis of the joint temperature, the joint temperatures at edge damage positions 2, 3, and 7 are all lower than those of the internal damage, but the average equivalent stresses are all greater than those of the internal damage, especially at position 2, which is at the edge tip; 6 × 107 Pa was the average stress. Although the edge damage is not on the primary heat flow path, it seriously jeopardizes the integrity of the interface, concentrating stress in the edge area. Furthermore, edge damage has a stronger propagation and is therefore more likely to result in large-area damage like interface delamination and cracking [27]. Nonetheless, in order to increase the yield rate, greater attention should be given to edge damage during the production process because it is relatively simple to detect on the chip [28,29].

**Figure 6:**
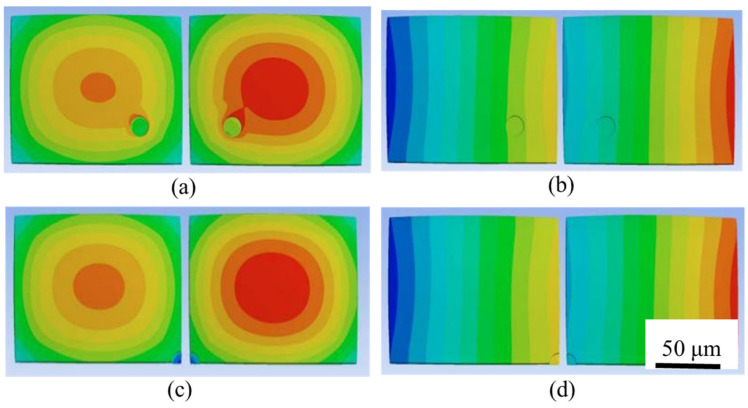
Distribution of thermal strain and x-axis directional deformation in the solder layer: (**a**) thermal strain distribution at position 5; (**b**) x-axis directional deformation at position 5; (**c**) thermal strain distribution at position 2; (**d**) x-axis directional deformation at position 2.

Table 2 provides an overview of the thermal strain, x-axis directional deformation, and total deformation at seven distinct positions, allowing for a more thorough investigation of the solder layer’s stress variation patterns. Upon examination of the data, it is evident that the patterns of strain and deformation align with the earlier analysis of stress and temperature. Because the solder layer’s material encloses the internal cavity, it is more restricted during thermal cycles, which prevents thermal expansion and raises the concentration of thermal stress surrounding the cavity [8,22]. The edge voids, due to the presence of more free surfaces nearby and less constraint, have lower thermal strain compared to the internal voids’ thermal stress. However, it is precisely because of this that symmetry does not constrain the deformation in the edge region, and during thermal cycling, the material close to the voids will undergo significant directional deformation. The solder layer’s edge has more voids than its interior, which results in the maximum and minimum differences in the x-axis directional deformation. Figure 6 illustrates this best by contrasting the experimental data at positions 2 and 5. In conclusion, the positions of various voids in the solder layer influence thermal strain and directional deformation in ways that are unique. The specific effects of voids on thermal stress must be carefully considered in thermal management and structural design, and damage monitoring must be improved.

Based on the above experimental data, it can be concluded that the gap at the edge of the solder layer can reduce the junction temperature, but at the same time, it will increase mechanical stress and failure risk. Therefore, we introduce a thermal/mechanical trade-off discussion or performance index to clarify the design optimization schemes under different priorities.

When conducting a thermal/mechanical trade-off analysis, it is necessary to quantitatively evaluate thermal performance (such as junction temperature) and mechanical performance (such as stress, failure risk). Based on thermodynamic and mechanical principles, to balance the conflicting trends in the design, the following three key parameter calculation formulas are introduced:

(1) Temperature reduction amplitude at the junction (Δ*T_j_*):

This describes the influence of edge voids on the junction temperature. The calculation formula is

(4) $$\Delta T_{j}=\frac{Q_{heat}}{h\cdot A}-\frac{Q_{heat}}{h_{new}\cdot A_{new}}$$

where Q_heat_ represents the heat flux density (W/m^2^), indicating the heat generated by the equipment. h is the original heat conduction coefficient (W/m^2^·K), reflecting the heat dissipation efficiency. hnew is the new heat conduction coefficient after introducing the edge voids. A and Anew are the heat dissipation areas of the original design and the new design (m^2^), respectively. This formula simulates the change in junction temperature through the change in heat flow path, and the larger the ΔT_j_ value, the more significant the temperature reduction effect.

(2) Mechanical stress increase amplitude (Δσ):

To assess the stress concentration caused by the edge voids, the calculation formula is

(5) $$\Delta \sigma =\sigma_{new}-\sigma_{original}=\frac{F_{load}}{A_{eff}}\cdot K_{t}$$

where *σ*_new_ and *σ*_original_ represent the stress levels of the new design and the original design (Pa). *F*_load_ is the external load (N), such as vibration or pressure. *A*_eff_ is the effective bearing area (m^2^), and the edge voids will reduce this area. *K_t_* is the stress concentration coefficient, which reflects the local stress amplification effect caused by the voids. The larger the Δσ value, the higher the risk of mechanical failure.

(3) Failure risk probability (*P_f_*):

Taking into account the influence of comprehensive junction temperature and stress, the possibility of failure is estimated. The calculation formula is as follows:

(6) $$P_{f}=f(\Delta T_{j},\Delta \sigma )=\frac{1}{1+e^{-k(\Delta T_{j}-\alpha \Delta \sigma )}}$$

where *f* represents the logical function, which maps the thermal mechanical parameters to probabilities. *k* and *α* are weight coefficients, representing the relative importance of temperature drop and stress increase, respectively. The closer the *P_f_* value is to 1, the higher the failure risk is, and it needs to be dealt with first.

To quickly determine the design priorities, the Δ*T_j_* and Δ*σ* values are calculated based on the above key parameter calculation formulas, and *P_f_* is derived. Finally, a trade-off decision is made by comparing the thresholds. The thermal/mechanical trade-off analysis for the solder layer is not the only application scenario of this type of collaborative optimization approach in power devices. This ‘simultaneous balancing of conflicting performance indicators’ design paradigm has also been verified at the core structure level of the devices [30].

### 3.2. Simulation Results of Four Typical Void Types

In this study, ANSYS Workbench software was used to perform a multi-void thermodynamic coupled analysis of the solder layer of an IGBT to investigate the effect of the type of void distribution on the thermal behavior of the IGBT. The four typical void distribution patterns selected by this simulation are random distribution, marginal distribution, uniform distribution, and centralized distribution. These patterns were chosen based on the commonality of IGBTs in real manufacturing processes and their representative potential impact on device performance. Random distribution simulates the voids generated during production due to material inconsistencies or process fluctuations. Marginal distribution reflects the scenario where voids tend to accumulate at the wafer’s edges due to edge effects or packaging stress. Centralized distribution considers the voids mainly gathering in the central area of the wafer, which is the region where thermal stress is most concentrated and fatigue accumulation is most pronounced.

**Figure 7:**
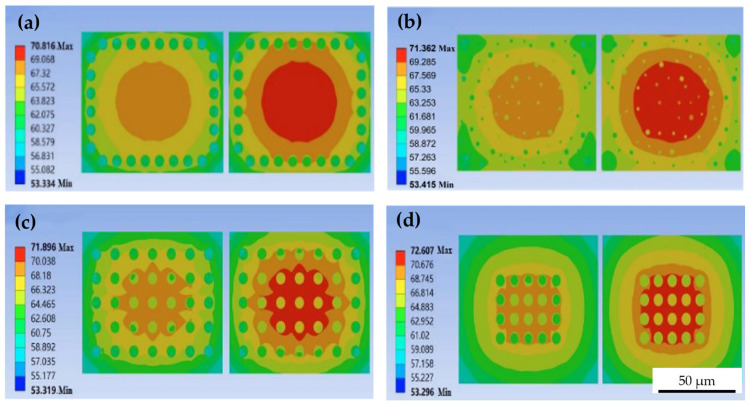
Temperature distribution cloud diagram of the solder layer under four types of cavity distribution: (**a**) marginal distribution type; (**b**) uniform distribution type; (**c**) random distribution type; (**d**) centralized distribution type.

Figure 7 shows the simulation results of the solder layer under four types of void distributions. The results indicate that damage with random distribution has the greatest impact on junction temperature, with the highest solder layer junction temperature reaching 73.5 °C, and the overall junction temperature reaching 73.4 °C, with the lowest junction temperature exceeding 68 °C, surpassing all other damage simulations.

A more thorough examination of the junction temperature and heat flux at the damage points reveals that dense damage points result in increased heat accumulation and uneven heat diffusion, which intensifies the thermal coupling effect [31,32,33]. The concentrated high-temperature area of the module with concentrated void distribution corresponds to a high power density region, although the junction temperature of the module is about 2 °C higher than it would be under normal circumstances. Due to the void aggregation, the solder exhibits localized inefficiency in heat conduction, which increases the risk of failure, severely damages the surrounding material, and creates a highly uneven junction temperature distribution. Because the damage is located further from the heat source center in the cases of uniform distribution and marginal distribution, the heat dissipates by the time it reaches the edges, having less of an effect on the junction temperature overall. On the other hand, asymmetric thermal stress distribution in the solder layer may result from the extensive damage, which could compromise the module’s mechanical integrity over time.

**Figure 8:**
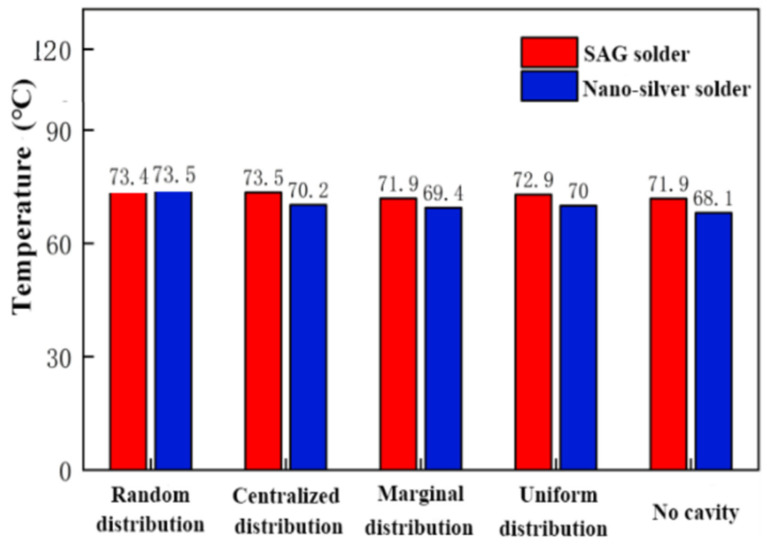
Simulation results of junction temperature using nano-silver solder and Sn-Ag-Cu solder modules under four types of damage.

To validate whether the aforementioned results are influenced by different types of solder, a similar simulation experiment was conducted using nano-silver solder, while keeping the simulation parameters unchanged. The results, as depicted in Figure 8, demonstrate that both solder types exhibit comparable temperature variation trends across different damage scenarios. However, due to the inherently higher thermal conductivity and lower thermal resistance of nano-silver solder, the overall junction temperature of the module was consistently lower in all four damage conditions compared to the module utilizing Sn-Ag-Cu solder. This corroborates the critical importance of damage detection and reliability assessment in the production and operation of IGBT modules.

**Figure 9:**
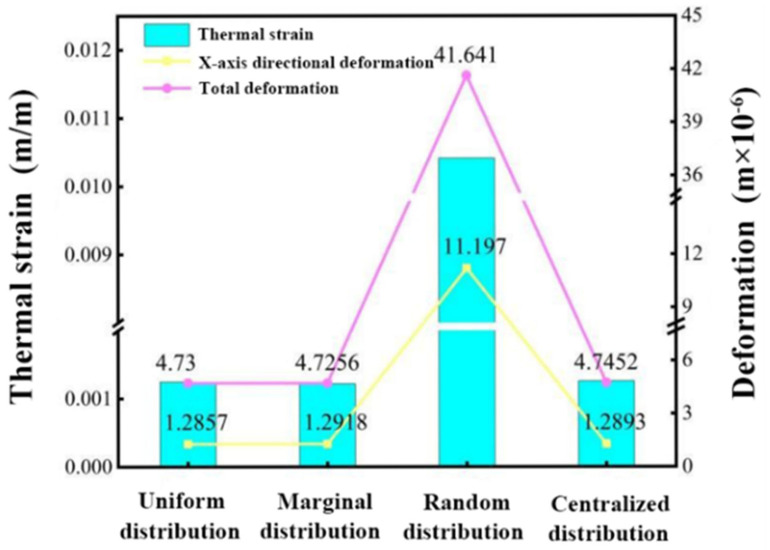
Simulation results of four types of damage stress.

**Figure 10:**
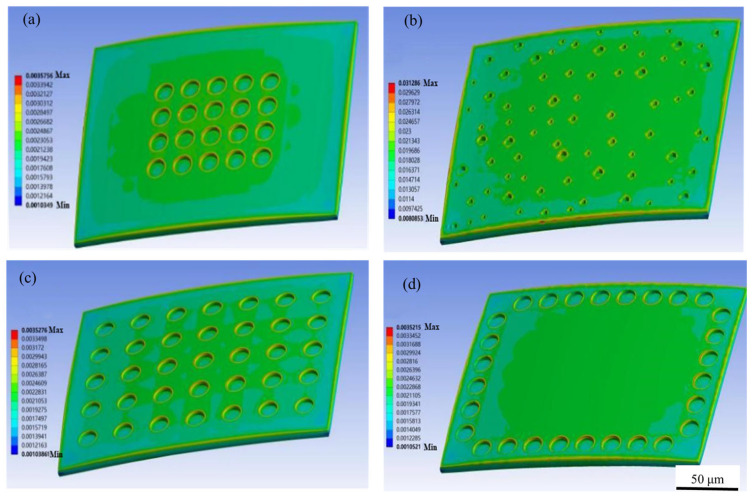
Elastic strain of the different solder layer types: (**a**) centralized distribution; (**b**) random distribution; (**c**) uniform distribution; (**d**) marginal distribution.

Table 3 provides a statistical analysis of the effects of four different damage types on the equivalent stress in the solder layer. The stress distribution pattern is significantly influenced by the void distribution. These voids also alter the load transmission paths, increasing the complexity of stress pathways, which results in higher localized stress in specific areas [34,35]. This leads to greater lateral and overall deformation, increasing the risk of damage propagation. Although the numerical difference between concentrated and uniform void distributions is small, the stress concentration effects vary significantly, as illustrated in Figure 9. Concentrated voids, due to widespread damage over a small area, exhibit the most pronounced stress concentration near the heat source. Furthermore, the elastic strains of different weld layer types were simulated and analyzed; the results are shown in Figure 10. These regions of high strain concentration reduce material strength around these clusters, increasing the risk of fatigue and fracture. In contrast, uniformly distributed voids diffuse the potential stress concentration effects across the entire solder layer, resulting in a relatively higher overall structural strength. Although the edge-distributed voids are located away from the heat source and exhibit the lowest stress values, the deformation along the x-axis is largest at the edges. This makes cracks more likely to propagate along the edges or from the edges toward the interior [36]. In conclusion, different types of void distributions have varying effects on the mechanical properties and reliability of the solder layer, which necessitates careful consideration in the design and evaluation processes.

The numerical simulation in this article regarding the thermal fatigue failure of the solder layer in IGBT modules utilizes the accelerated life test algorithm and multi-physics field coupling technology to significantly enhance the time efficiency. By constructing a parametric model using ANSYS for cyclic loading simplification, it saves approximately 80% of the time cost compared to traditional experimental methods. Under typical conditions, the single thermal cycle simulation time is reduced from 72 h to 15 h, and parallel computing can achieve synchronous simulation of multiple working conditions. This digital approach effectively avoids the production cycle of physical prototypes, increasing the failure prediction efficiency during the research and development stage by 3–4 times, providing an efficient analysis strategy for the reliability optimization of power modules.

Based on the aforementioned simulation setting of the experimental parameters, a thermal cycling experiment was conducted on the solder layer of the IGBT module, and then a microscopic structure analysis was carried out. The results are shown in reference [33].

The multi-performance trade-off optimization of existing power devices has gradually expanded from the single device dimension to the collaborative design of the entire chip-packaging chain. The latest research by Feng et al. [37] achieved decoupling of multiple physical parameters within SiC devices through an embedded MOS structure, significantly improving the high-voltage and high-frequency performance without increasing manufacturing costs. This innovative stress redistribution at the device level also poses new requirements for the solder layer design at the packaging end. This paper, based on the void location thermal stress evolution law obtained from traditional IGBT modules, can precisely provide a universal analytical approach for the packaging optimization of these advanced SiC devices, filling the research gap between device innovation and packaging adaptation.

The current single thermal stress simulation model of this study has clear applicable boundaries, and its limitations mainly lie in three aspects: Firstly, it does not consider the electrochemical corrosion effect of the solder layer caused by the bias voltage, and thus cannot directly reproduce the accelerated aging process under the condition of electrical wiring. Secondly, it does not incorporate the self-heating effect of the chip caused by the conduction current, and the stress amplitude of the solder layer obtained from the simulation is lower than the measured value under the actual vehicle wiring condition. Thirdly, the solution logic for the coupling of electric, thermal and mechanical fields has not been established yet, and it can only be used for the prediction of basic fatigue laws in passive thermal cycling scenarios. In the future, the model will be iterated by introducing an electric stress coupling module.

## 4. Conclusions

To investigate the impact of crack size, location, and irregular distribution on the module’s thermal properties, a total of 37 solder layer damage models were created. Changes in pertinent parameters that resulted from various damage conditions for IGBT modules were obtained. The highest stress at the edge of the solder layer is 6.2504 × 10^7^ Pa, the lowest junction temperature is 70.79 °C, and the average thermal stress is 1.2388 (m/m). The highest junction temperature can reach 72.256 °C under various solder layer damage states by a thermal/mechanical coupled simulation analysis of four different types of solder layer defects. According to the simulation results, there is a linear relationship between the void ratio and junction temperature in the range of 5–20%. Larger void sizes result in noticeably higher temperatures and thermal resistance, indicating that to preserve module performance, the void ratio should be kept below 3%. Furthermore, while the junction temperature variation rate is more significantly impacted by the size of through-thickness voids, non-through-thickness voids have a greater effect on changing the module’s heat dissipation path. Stress is increased when there is internal solder layer damage, whereas strain is increased when there is edge damage. The stress concentration brought on by different types of solder damage was also found to differ significantly in this study, with the stress concentration being more noticeable at the edges surrounding the solder layer. The reason for this result is that the edge damage is not on the main heat flow path, but it causes significant damage to the interface integrity, resulting in stress concentration in the edge area. Moreover, the edge damage has greater expansibility and is more prone to causing large-scale damage, such as interface peeling and cracking, which is the main cause of fatigue failure.

## Figures and Tables

**Table 1:** Material parameters of IGBT modules.

Materials	Density Kg/m^3^	Thermal Conductivity W/(m·K)	Thermal Expansion Coefficient 10^−6^ k^−1^	Poisson’s Ratio	Specific Heat Capacity J/(kg·k)	Young’s Modulus MPa
Cu	8600	390	17	0.37	390	110,000
Sn-Ag-Cu	7300	54	25	0.4	230	34,300
AlN	3400	320	4.5	0.22	710	310,000
Al	2690	119	2.99	0.28	700	167,000
Nano silver	8500	160	19.5	0.25	235	50,000
92.5 Pb 5 Sn 2.5 Ag	11,000	35.8	29	0.35	170	24,700
Air	1.09	2.733	-	-	1.017	-
Sn 63 Pb 37	8400	51	25	0.189	150	28,000
Sn-Cu 0.7	7310	80	23	0.37	220	30,000

**Table 2:** Thermal strain and deformation of the solder layer corresponding to different void damage locations [9].

Position Number	Thermal Strain (m/m)	X-Axis Directional Deformation Max (m)	X-Axis Directional Deformation Min (m)	Total Deformation (m)
1	1.2642	1.2964 × 10^−6^	−1.2474 × 10^−6^	4.7316 × 10^−6^
3	1.2199	1.3026 × 10^−6^	−1.2536 × 10^−6^	4.7280 × 10^−6^
5	1.2278	1.2969 × 10^−6^	−1.2479 × 10^−6^	4.7286 × 10^−6^
6	1.2434	1.3023 × 10^−6^	−1.2531 × 10^−6^	4.7282 × 10^−6^

**Table 3:** Equivalent stress of the solder layer for four types of damage.

Type of Damage	Maximum Equivalent Stress (Pa)	Minimum Equivalent Stress (Pa)
Centralized distribution	8.2924 × 10^7^	2.2605 × 10^7^
Random distribution	7.1908 × 10^8^	1.8167 × 10^8^
Uniform distribution	8.3005 × 10^7^	2.2681 × 10^7^
Marginal distribution	8.179 × 10^7^	2.2797 × 10^7^

## Data Availability

The original contributions presented in this study are included in the article; further inquiries can be directed to the corresponding author.
